# Oxidative Model of Retinal Neurodegeneration Induced by Sodium Iodate: Morphofunctional Assessment of the Visual Pathway

**DOI:** 10.3390/antiox12081594

**Published:** 2023-08-10

**Authors:** Michael D. Espitia-Arias, Pedro de la Villa, Victor Paleo-García, Francisco Germain, Santiago Milla-Navarro

**Affiliations:** 1 Department of Systems Biology, University of Alcalá, 28805 Madrid, Spain; michael.espitia@edu.uah.es (M.D.E.-A.); pedro.villa@uah.es (P.d.l.V.); victor.paleo@edu.uah.es (V.P.-G.); 2 Visual Neurophysiology Group-Instituto Ramón y Cajal de Investigación Sanitaria (IRYCIS), 28034 Madrid, Spain

**Keywords:** *Opn4*, oxidative stress, sodium iodate, neurodegeneration

## Abstract

Sodium iodate (NaIO_3_) has been shown to cause severe oxidative stress damage to retinal pigment epithelium cells. This results in the indirect death of photoreceptors, leading to a loss of visual capabilities. The aim of this work is the morphological and functional characterization of the retina and the visual pathway of an animal model of retinal neurodegeneration induced by oxidative stress. Following a single intraperitoneal dose of NaIO_3_ (65 mg/kg) to C57BL/6J mice with a mutation in the *Opn4* gene (*Opn4^−/−^*), behavioral and electroretinographic tests were performed up to 42 days after administration, as well as retinal immunohistochemistry at day 57. A near total loss of the pupillary reflex was observed at 3 days, as well as an early deterioration of visual acuity. Behavioral tests showed a late loss of light sensitivity. Full-field electroretinogram recordings displayed a progressive and marked decrease in wave amplitude, disappearing completely at 14 days. A reduction in the amplitude of the visual evoked potentials was observed, but not their total disappearance. Immunohistochemistry showed structural alterations in the outer retinal layers. Our results show that NaIO_3_ causes severe structural and functional damage to the retina. Therefore, the current model can be presented as a powerful tool for the study of new therapies for the prevention or treatment of retinal pathologies mediated by oxidative stress.

## 1. Introduction

Science has generated animal models that can mimic the visual damage present in many human diseases [1,2]. Thanks to them, progress has been made in understanding the mechanisms that produce such damage and in the design of treatments. Currently, oxidative damage is a major focus of research. For an animal model to be useful, it is necessary to know the type and intensity of the damage caused by a given lesion stimulus, so it must be comprehensively evaluated.

The visual pathway is frequently used to study different types of central nervous system lesions due to its experimental accessibility. The pathway starts with phototransduction by the classical photoreceptors, cones and rods, and by the intrinsically photosensitive Retinal Ganglion Cells (ipRGCs) of the inner retina. Whereas the former is responsible for image formation, the latter is involved in other functions such as the regulation of circadian cycles [3,4,5,6] and the pupillary reflex [3], among others. The photosensitivity of the ipRGC is due to the presence of melanopsin, a protein belonging to the opsin family, which is encoded by the *Opn4* gene [3].

Closely related to the outer segments of the photoreceptors is the retinal pigment epithelium (RPE), which is involved in the photoreceptor maintenance and protection and participates in the re-isomerization of the all-trans retinal complex [7]. Damage to this epithelium could impair visual function. Examples of this can be found in diseases such as Retinitis Pigmentosa, Leber’s Congenital Amaurosis, Stargardt’s syndrome, and age-related macular degeneration (AMD) in its two variants [8,9,10,11].

Having a stable model of RPE degeneration by oxidative stress would provide a very useful tool for studying the effects and mechanisms of action of potential antioxidants. In this sense, the oxidative damage produced after the administration of sodium iodate (NaIO_3_) is well known [12,13,14,15,16,17,18]. Since the first studies in 1941 [19], various experiments have been performed to characterize this process. The importance that this murine model of NaIO_3_ injury has acquired today is based on the fact that, with new high-resolution in vivo study techniques (spectral domain optical coherence tomography (OCT) and confocal scanning laser ophthalmoscopy (cSLO)), it is possible to analyze the retinal degeneration from a histological point of view [14,20].

Several important aspects have been identified in relation to retinal damage by sodium iodate. The first of these is oxidative stress, which acts by selectively damaging the RPE in a manner like age-related macular degeneration (AMD). However, the role of Reactive Oxygen Species (ROS) in NaIO_3_-stimulated signaling pathways and cell viability has not been fully elucidated. The effect of NaIO_3_ on autophagy in RPE cells also remains elusive. It is known that oxidative stress produced by NaIO_3_ results in cytosolic ROS production, which, through multiple coordinated signaling pathways, controls RPE cell death [21].

Systemic administration of NaIO_3_ at high doses damages the RPE by oxidative stress, leading to the loss of photoreceptors. The reaction of NaIO_3_ with melanin increases toxicity through the transformation of glycine to glyoxylate and inhibits or alters enzymes related to cellular energetics [12,13,14,15,16,17,18]. On the other hand, NaIO_3_ disrupts the interaction of the RPE with photoreceptor outer segments and destroys the blood–retinal barrier, thus losing its homeostasis and nutrient delivery mechanisms. This destruction causes macrophages to accumulate in the damaged areas of the retina and release factors that will affect the structure of the photoreceptors [22]. The mechanisms of death caused by NaIO_3_ include necrosis and necroptosis [18,23]. Thus, it has been observed that at lethal doses, NaIO_3_ causes the death of RPE cells that involve executional caspase 3/7/8-dependent apoptosis and caspase-independent cell necroptosis. This RPE lesion was subsequently responsible for inducing photoreceptor cell death [24,25]. In addition, a local immune response manifested by the elevation of markers of inflammation in the retina has been observed. Thus, it is reasonable to think that macrophages, microglia, and gliotic Müller cells may be a source of local inflammatory changes in the retina. In contrast, systemic complement and cytokines/chemokines did not undergo changes in this model of retinal degeneration after NaIO_3_ administration [26,27].

Whereas some resistance to peripheral retinal damage has been observed at low doses, at high doses, the damage results in massive degeneration of photoreceptors throughout the retina. Furthermore, some studies have reported that NaIO_3_ causes damage beyond the photoreceptors, based on the absence of immunohistochemical labeling of bipolar rod cells by anti-PKCα (protein kinase C alpha) antibodies [28].

For the assessment of the functional impact of oxidative damage on cones and rods, responses from ipRGCs should be excluded, as they could lead to a bias in the interpretation of the results [29,30,31]. Such exclusion can be achieved by deletion of the *Opn4* gene (*Opn4^−/−^*), which prevents the expression and synthesis of melanopsin in the ipRGCs and thus their function [32,33,34]. Thus, we can ensure that upon application of an antioxidant molecule in this injury model, any favorable response can be attributed to that molecule and not to a residual function of other photosensitive cells remaining after injury to the classical photoreceptors [32,33,34].

We hypothesized whether we would be able to generate a model of photoreceptor oxidative injury that could be used to functionally assess the response to antioxidants. Therefore, the main objective of our work was to characterize an animal model whose photoreceptors had been damaged by the oxidizing agent NaIO_3_, so that the location and degree of damage would be perfectly known. A perfect description of this model is essential to be able to test new avenues of research focused on the preservation of visual functions in the face of oxidative damage. For this purpose, we have extensively characterized functionally, structurally, and behaviorally an animal model of oxidative damage in the visual pathway, generated by the systemic application of NaIO_3_ in a strain of mice with a mutation that inhibits melanopsin synthesis (*Opn4^−/−^*). The aim was to verify that the oxidative model met the necessary requirements to consider its blindness as absolute. Thus, the absence of the photoreceptors involved was demonstrated by immunohistochemical techniques. The functional alteration of these photoreceptors, as well as the affectation of the visual cortex, were analyzed by means of electrophysiological tests. Finally, visual acuity was measured, and behavioral tests were performed.

## 2. Materials and Methods

### 2.1. Animal Housing

The experimentation and scientific procedures carried out followed the European regulations (Directive 86/609/CEE) and national (Royal Decree 53/2013, 1 February) laws that protect animals used for experimentation and other scientific purposes, as well as Law 32/2007 for the care of animals in their exploitation, transport, experimentation, and sacrifice. In addition, the guidelines of the Association for Research in Vision and Ophthalmology (ARVO) were adhered. All experiments involving the use of experimental animals were approved by the Ethics Committee of the University of Alcalá (CEI-UAH-AN2016008//PROEX 147 and CEI-UAH-AN2021009 PROEX 147).

Twenty-six mice (*Mus Musculus*) of 10–12 weeks of age were used. The animals were distributed homogeneously by gender (50% females and 50% males) in three different groups to sufficiently space the anesthetic administration and to avoid possible alterations of the results due to anesthetic procedures. Mice were housed in ventilated cages with High-Efficiency Particulate Air Filters (HEPA) and exposed to a 12 h light (20 ± 1 Luxes) and 12 h dark cycle. The room had an ambient temperature of 21 °C and a relative humidity of 55 ± 10%. Mice were allowed to eat and drink ad libitum. Weekly cleaning was carried out. To compare with the experimental animals, C57Bl/6J mice with a deletion mutation of the *Opn4* gene (*Opn4^−/−^*), were used as a control.

### 2.2. Anesthesia

The anesthesia protocol was applied to all experiments except for the records of the pupillary reflex. The animals were weighed and anesthetized using a ketamine anesthetic solution (Ketamidor, Richter Pharma AG, Wels, Austria) (100 mg/mL), xylazine (Xilagesic, CALIER, Barcelona, Spain) (20 mg/mL), and NaCl (Grifols, Barcelona, Spain) (0.9%). Then, 0.5 mL/150 g of this compound was administered intraperitoneally with a 25 G needle.

For pupillary reflex analysis, animals were anesthetized with isoflurane (gas) (2%, Isoflo©, Zoetis S.L., Madrid, Spain), maintaining a constant flow of 0.4 L/min of O_2_ through a TEC 3 vaporizer (MSS International Ltd., Keighley, UK).

Once the experiments or surgeries were completed, the animals were helped to recover by leaving them on a thermal blanket.

### 2.3. Implantation of a Chronic Electrode

The chronic implantation of electrodes in the visual cortex of the animals was necessary to perform the double electrophysiological recording of Electroretinogram and Visual Evoked Potentials (VEP) [35,36,37,38]. The surgical material was previously disinfected with 70% ethanol. The animals were weighed and anesthetized before being placed in a stereotaxic apparatus (Stoelting, Wood Dale, IL, USA). After cutting the hair between the ears and cleaning and disinfecting the entire surface, a longitudinal cut was made in the skin just between both ears, and the connective tissue was removed by scraping with a scalpel. A hole was drilled in a very precise place in the skull (V1, 0.61 mm rostral from Lambda and 2.3 mm left lateral) using a hand drill with a stainless-steel drill bit (0.8 mm diameter) (Dremel Multipro, Madrid, Spain). Taking advantage of this hole, a stainless-steel screw (M1-4 mm) was placed in the hole, so that the base was in contact with the surface of the brain, but without entering the visual cortex. To promote proper screw fixation and healing, the wound edges were brought together, and the area was covered with dental resin (DuraLay, Reliance, IL, USA). To treat pain, the analgesic (Meloxidyl, Veterinaria Esteve, Barcelona, Spain) was dissolved in drinking water at a dose of 5 mg/100g/day for 24 h before the surgery. Recovery of the mouse without signs of pain or agony was achieved by avoiding any manipulation until 48 h after the intervention.

### 2.4. Light/Dark Transition Test

In a box 59 cm high and 28.5 cm × 28.5 cm on each side, two compartments, one illuminated and one in darkness, were differentiated. Both compartments were connected by a small opening, which was blocked when the mouse was initially placed in the box, thus preventing its escape [39]. In the illuminated box, an LED strip (Ledflexi Inspire, Leroy Merlin, France) provided a light intensity of 484.3 Lux, while only 0.21 Lux was measured in the dark box. A few seconds after the animal was placed in the box, the passage was unlocked, allowing free movement between the two compartments. The time spent in each compartment was timed at 3 min. The animal’s body had to pass completely through the opening to be considered as a change of compartment. After repeating this operation three times, the results were averaged.

### 2.5. Optomotor Test

The stimulation was produced by 4 screens (FLATRON, LG, Seoul, Republic of Korea) facing each other two by two, closing the quadrilateral. A transparent methacrylate cylinder was placed in the center of the quadrilateral, and the mouse was placed inside it so that its movement was reduced, which promoted the animal’s response to the stimulation. The visualization of its movements was performed by means of an infrared camera (AVC-D5CE, SONY, Tokyo, Japan) placed through a small opening in the upper part.

The stimulation consisted of a series of black and white vertical bars occupying the entire screen and moving sideways. These bars had different spatial frequencies (0.011, 0.022, 0.044, 0.088, 0.177, and 0.355 cycles per degree, cpd) and different contrasts (100, 50, 25, 10, and 5%). The generated pseudo-rotational effect caused the mice to follow it with their head movement. After randomly presenting the stimuli for 20 s in both directions of rotation (clockwise and counterclockwise), to ensure greater objectivity, it was considered positive if the animal had moved its head in the same direction as the stimulus presentation. Three hits had to occur for each stimulus to ensure that the results were true.

### 2.6. Pupillary Light Reflex (PLR)

The anesthetized (isoflurane) mouse was placed under a laboratory magnifier (Wild Heerbrugg, Leica, Switzerland). After resting for 5 min to stabilize the pupil, it was stimulated with light for 1 min, after which it was returned to darkness for 2 min to return the pupil to the basal state. This protocol was repeated three times in a row per animal. Finally, the mouse was left for a few minutes to recover properly before being returned to its cage.

Pupillary area variation was observed using an infrared camera (AVC-D5CE, SONY, Tokyo, Japan) coupled to a laboratory magnifier. This device was placed 15 cm from the eye, and pupil photographs were taken every 10 s. The pupil area was measured using ImageJ image analysis software, version number 1.52p (U. S. National Institutes of Health, Bethesda, MD, USA) in its portable version (FIJI).

### 2.7. Full-Field Electroretinogram (ERG)

After 12 h in darkness, the mice were anesthetized under dim red light as the only illumination, while recordings were made in complete darkness. The mouse was placed inside a Faraday cage and on a warm blanket (T/Pump TPP522, Gaymar Industries, New York, NY, USA) that maintained the body temperature at 37 °C. The pupils were dilated with a drop of 1% Tropicamide (Alcon Cusí, SA, El Masnou, Barcelona, Spain). The grounding electrode was placed at the base of the tail, the reference electrode on the tongue, while a gold band electrode placed on the cornea was used for recording. To protect the cornea and improve signal conductivity, methylcellulose drops (2% Methocel, Omnivision, Neuhausen, Switzerland) were administered. The right eye responses produced after light stimulation by the Ganzfeld dome were first recorded under scotopic conditions at increasing intensities (−4.0, −3.0, −2.0, −1.5, −1.0, −0.5, 0.0, 0.5, 1.0, and 1.5 log cd·s·m^−2^), averaging at least 10 signals per stimulus and increasing the time between stimuli to avoid bleaching of the photoreceptors. In order to analyze the signals, they were amplified 1000 times using a NeuroLog System (NL900D NeuroLog System Rac, Digitamer Ltd., Letchworth Garden City, UK) and band-pass filtered between 0.3 and 1000 Hz. The electrophysiological signal was digitized with a Power Lab 4/30 data acquisition card (ADInstruments Ltd., Oxfordshire, UK) at 10 kHz. For recording under photopic conditions, the animals were adapted to light for 5 min at an intensity of 30 cd·m^−2^. Then, flashes of increasing intensity (−1.0, −0.5, −0.0, −0.0, 0.5, 1.0, and 1.5 log cd·s·m^−2^) and with lower inter-stimulus times were used for photopic recording. A stimulation frequency of 20 Hz and an intensity of 1.5 log cd·s·m^−2^ were used to record the flicker response. Commercial software LabChart Pro v.8.1.13 (ADInstruments Ltd., Oxfordshire, UK) was used to manually measure the amplitude of the ERG wave components and digitally analyze the oscillatory potentials, applying a 100 Hz high-pass filter for the 1.5 cd·s·m^−2^ intensity record.

### 2.8. Visual Evoked Potentials (VEP)

One week after electrode implantation, mice were anesthetized following the protocol described above and placed on a thermal blanket inside a Faraday box. A gold rod placed on the tongue of the animals was used as a reference electrode, which avoided implanting a second chronic electrode, but allowed robust recordings of the different components of the VEP. Grounding was performed by means of a needle placed at the base of the tail. VEP recording was performed using fine forceps connected to the electrode surgically implanted in the skull of the mouse.

VEP recording followed the same protocol as ERG recording by stimulation with an appropriately calibrated Ganzfeld dome. Each signal was averaged about 300 times, amplified 100,000 times, and filtered through a band-pass filter between 5 and 500 Hz. The amplification and filtering equipment used was NeuroLog System (NL900D NeuroLog System Rac, Digitamer Ltd., Letchworth Garden City, UK). The collected signal was digitized at 10 KHz using a Power Lab 4/30 data acquisition card (ADInstruments Ltd., Oxford, Oxfordshire, UK). Waveform analysis was performed manually using commercial software LabChart Pro v.8.1.13 (ADInstruments Ltd., Oxford, Oxfordshire, UK).

### 2.9. Inmunohistochemistry

Immediately after sacrifice of the mice by pentobarbital overdose (Dolethal, Picemar, Castellón, Spain), the eyes were enucleated and fixed with 4% PFA for 1 h 30 min. The lens was then removed, and the optic cup was kept for another 30 min in 4% PFA. Subsequently, they were cryoprotected by an increasing sucrose gradient (20% 1 h, 30% 1 h sucrose (*w/v*) in 0.1 M phosphate buffer, pH 7.4) and finally at 40% overnight. The next day, the optic cup was included in OCT (Optimal Cutting Temperature, Sakura Finetek, Torrance, CA, USA) and frozen at −20 °C until the samples were cut.

The optic cup was cut into 15 µm serial cross sections by cryostat (Leica CM1950, Leica, Switzerland). After collecting the sections, 3 washes of 10 min each were performed under agitation. When immunohistochemistry was started, a preincubation with 0.5% Triton X-100 in 0.1M PBS was performed to permeabilize the tissue. Retinal sections were incubated with 0.5% Triton X-100 in 0.1M PBS, 2% serum, and primary antibodies (concentration depends on the antibody used, Table 1A) overnight in a humid chamber. After the primary incubation, 3 washes of 10 min were performed, followed by incubation with the secondary antibodies (Table 1B), diluted in 0.5% Triton X-100, 0.1M PBS and 2% serum, for 1 h and 30 min. Subsequently, 3 new washes of 10 min each were performed, and a DNA intercalating marker (4′,6-diamidino-2-phenylindole (DAPI)) (Sigma/Merck KGaA, Darmstadt, Germany) was added at a concentration of 0.01%, which allowed the visualization of cell nuclei, its emission wavelength was 470 nm. Finally, after several washes, the sections were mounted with the appropriate medium. 

The number of nuclei per column, the total thickness of the retina (from the inner to the outer limiting membrane), and the relative thickness of the inner nuclear layer (INL) and the outer nuclear layer (ONL) layer were measured on sagittal slices made in the center of the eye to calculate the INL/total thickness and ONL/total thickness ratios. In this way, we ensured that the measured variation in the thickness of a layer was not due to oblique shearing.

### 2.10. Statistical Analysis

Data were statistically analyzed using GraphPad Prism 8.0 software (GraphPad Software Inc., La Jolla, CA, USA). Normality between groups was ensured by the Shapiro–Wilk test. When a variable was compared between two groups, a paired or unpaired Student’s *t*-test was performed depending on the characteristics of each comparison. For the analysis of several groups with more than two variables, two-way ANOVA and Sidak’s multiple comparison were used. In all cases, the statistical significance value was set as *p*-value < 0.05. If higher significance indices were obtained, they are appropriately indicated in the image by asterisks (*, *p* < 0.05; ** *p* < 0.01; *** *p* < 0.001; ns, no signification).

## 3. Results

### 3.1. Light/Dark Transition Test

**Figure 1:**
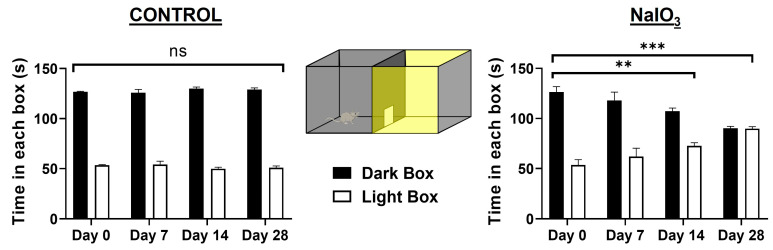
Average time spent by the animals in the control group and the experimental NaIO_3_-injected group in the compartments of the light/dark transition test at the different experimental times. Black and white histograms represent time spent in the dark and light compartment. The light sensitivity of the experimental group significantly decreased at 14 and 28 days. Data are presented as mean ± SEM. Significance studied with respect to day 0 (*n* = 10, *p* < 0.001), (ns, not statistically significant; **, *p* < 0.01; ***, *p* < 0.001).

This test allowed us to observe a progressive and significant decrease in the ability of the animals to detect illuminated spaces after NaIO_3_ injection at 7, 14, and 28 days, analyzing the mean time they remained in the dark compartment (see Figure 1). No significant differences were observed between the control group and the experimental group prior to injury (day 0) (*n* = 10, *p* > 0.999). There were significant differences between day 0 and day 14 after injection in the injured group (*n* = 10, *p* = 0.001). Finally, after 28 days, the loss of light sensitivity was accentuated (*n* = 10, *p* < 0.001). By day 28, animals were unable to recognize the illuminated compartment, spending approximately 50% of the time in each compartment.

### 3.2. Optomotor Test

This test measures the visual acuity of the animals by observing the movement of the animal’s eyes/head following the direction of rotation of the grating stimuli [40]. The spatial frequency, direction, and contrast of the stimuli were varied to determine visual acuity.

**Figure 2:**
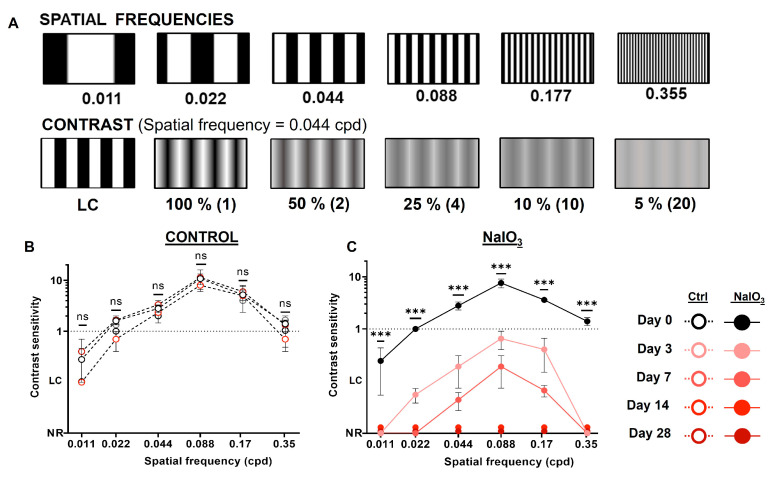
Visual acuity of animals injected with NaIO_3_ at different experimental times. (**A**) Representation of different spatial frequencies (top row, cycles per degree—cpd) and linear contrast (LC) or sinusoidal stimuli (100–5%, bottom row) applied in the optomotor test. Contrast sensitivity (inverse of contrast percentage) is indicated in brackets at the left of the contrast. (**B**) Evolution of the visual acuity of the control group. No significant differences (ns) were observed at different days. (**C**) Evolution of the visual acuity of the experimental group (NaIO_3_). The visual acuity of the animals decreased significantly at 3 and 7 days compared to day 0. However, some animals still responded to LC. By day 14, the animals were not able to perceive any stimuli (No Response, NR) (*n* = 8, *p* < 0.001). Data are presented as mean ± SD, (ns, not significant; ***, *p* < 0.001).

The injection of NaIO_3_ caused a decrease in the visual acuity of the animals (see Figure 2). Prior to the induction of retinal damage (day 0), the animals showed standard visual acuity for the various spatial frequencies and contrasts of the sinusoidal gratings used (contrast sensitivity above 1), with no difference compared to the control group (*n* = 8, *p* = 0.1097). Visual acuity was severely impaired 3 days after NaIO_3_ injection (*n* = 8, *p* < 0.001) and then 7 days later (*n* = 8, *p* = 0.015, relative to day 3), since it was necessary to use high-contrast black/white stripes to elicit the optomotor response. From 14 days after administration, the animals were unable to detect any of the stimuli presented. 

### 3.3. Pupillary Light Reflex (PLR)

**Figure 3:**
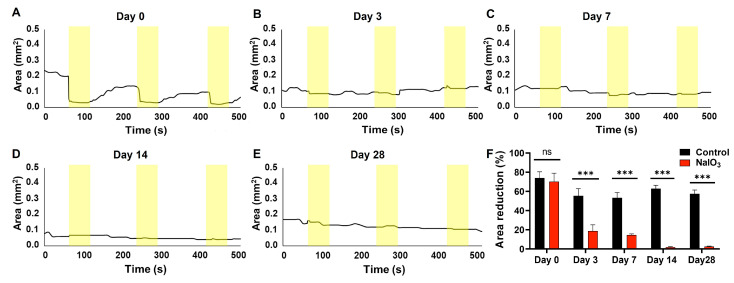
Pupillary light reflex after NaIO_3_ administration (Day 0, (**A**); Day 3, (**B**); Day 7, (**C**); Day 14 (**D**); Day 28, (**E**)). (**F**) Mean percentages of area reduction at different time points (*n* = 10). NaIO_3_-induced injury resulted in a near total loss of pupillary reflex after 3 days (*n* = 8, *p* < 0.001). Area was measured in mm^2^. Data are presented as mean ± SEM, (ns, not significant; ***, *p* < 0.001).

This test measures the pupillary contraction of the animals to a light stimulus [40]. It was observed that NaIO_3_ injection significantly decreased pupillary contraction, manifesting as a smaller change in the pupillary area to three consecutive stimuli (see Figure 3). Significant differences were observed 3 days after administration compared to the the control group (*n* = 8, *p* < 0.001). The pupillary reflex was virtually inhibited 14 days after administration.

### 3.4. Full-Field Electroretinogram

**Figure 4:**
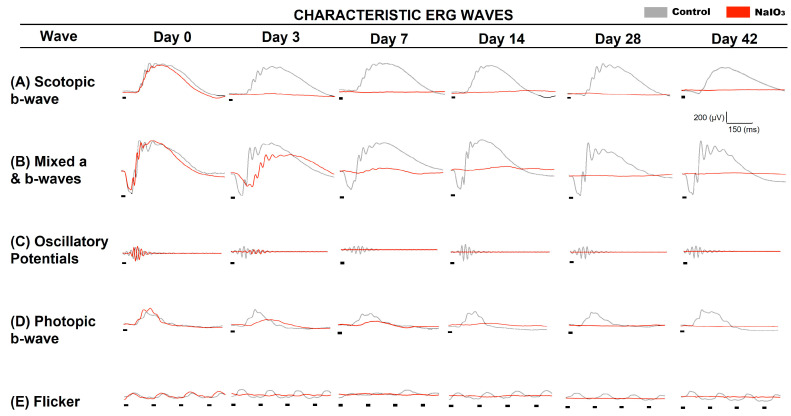
Electroretinographic responses after NaIO_3_ injection. Electroretinographic recordings from an animals injected with NaIO_3_ (red traces) compared to a control animal (dark traces). (**A**) Rod response under scotopic conditions (b-scot, −1 log cd·s·m^−2^). (**B**) Mixed rod and cone response under scotopic conditions (a and b-mix, 1.5 log cd·s·m^−2^). (**C**) Oscillatory potential (OP) recording under scotopic conditions (1.5 log cd-s-m^−2^). (**D**) Cone response under photopic conditions (b-phot, 1.5 log cd·s·m^−2^). (**E**) Flicker response of cones under photopic conditions (1.5 log cd·s·m^−2^). There was a near total decrease in the amplitude of all ERG waves 14 days after sodium iodate administration. Red trace (mice injected with NaIO_3_) (*n* = 10), gray trace (control mice) (*n* = 10). The black lines under traces represent the light stimulus.

Using the full-field ERG technique, retinal functionality was recorded at different experimental times to study the evolutionary course of neurodegeneration after NaIO_3_ administration (see Figure 4).

**Figure 5:**
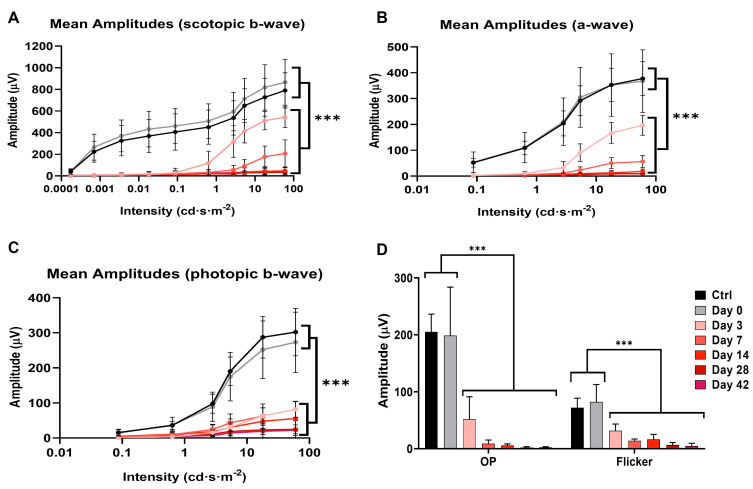
Variation of b- and a- wave amplitude under different light conditions, recorded for each of the experimental times after NaIO_3_ administration (Control, *n* = 10; NaIO_3_, day 0, *n* = 10; NaIO_3_, day 3, *n* = 8; NaIO_3_, day 7, *n* = 8; NaIO_3_, day 14, *n* = 10; NaIO_3_, day 28, *n* = 10). (**A**) b-wave and (**B**) a-wave under dark-adapted conditions. (**C**) b-wave under light-adapted conditions. (**D**) Representative histogram of the amplitude of the Oscillatory Potentials (OP) and flicker. Data are presented as mean ± SD (***, *p* < 0.001). The results obtained both in the control and in the different days after the injection of sodium iodate are reflected in the color code of the attached legend.

Analysis of the different wave amplitudes recorded in the ERG showed statistically significant differences between control and NaIO_3_-injected animals (see Figure 5). Three days after NaIO_3_ injection, the five ERG characteristic waves (scotopic b-wave and a-wave, Oscillatory potentials (OP), photopic b-wave, and flicker) of the impaired group were significantly decreased (*n* = 8, *p* < 0.001) relative to the control. Subsequently, the amplitude of ERG waves continued to decrease at 7 and 14 days (*n* = 10, *p* < 0.001), and it became almost impossible to record any light response after this time *p*. Thereafter (14, 28 and 42 days), no significant differences were observed in the scotopic b-wave (bipolar rod cell response) (*n* = 10, *p* = 0.215). However, significant differences in the a-wave (*n* = 10, *p* = 0.002) and photopic b-wave (*n* = 10, *p* < 0.001) were observed between these days, suggesting that cones were degenerating more slowly.

### 3.5. Visual Evoked Potentials

**Figure 6:**
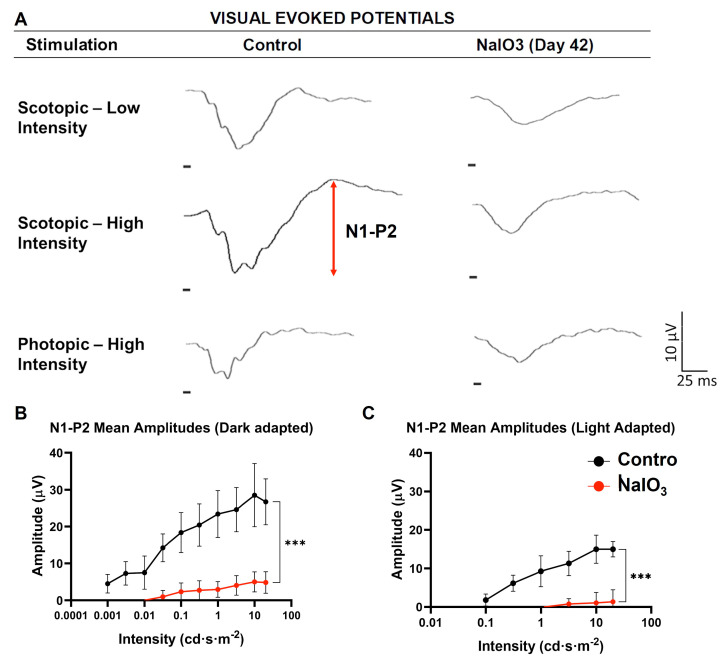
Effect of NaIO_3_ injection on visual cortex activity. (**A**) Recordings of Visual Evoked Potentials under scotopic conditions with low- and high- intensity stimulation, as well as under photopic conditions. Arrow represents N1−P2 amplitude. (**B**) Recording of N1−P2 amplitude as a function of the applied light intensity under dark-adapted conditions. (**C**) Recording of N1−P2 amplitude as a function of the applied light intensity under light-adapted conditions (***, *p* < 0.001).

Recordings of Visual Evoked Potentials (VEP) made it possible to study the activity of the primary visual cortex of these animals in response to light stimulation of the retina. In this way, the state of the visual pathway could be ascertained (see Figure 6).

In the NaIO_3_-administered animals, characteristic VEP waves were recorded, showing a decrease in amplitude compared to the control group. Large statistical differences in N1−P2 amplitude were observed in both scotopic and photopic conditions (*n* = 10, *p* < 0.001). These results suggest that there was still a small amount of photosensitive activity remaining in the retina, which was amplified along the visual pathway.

### 3.6. Inmunohistochemistry

**Figure 7:**
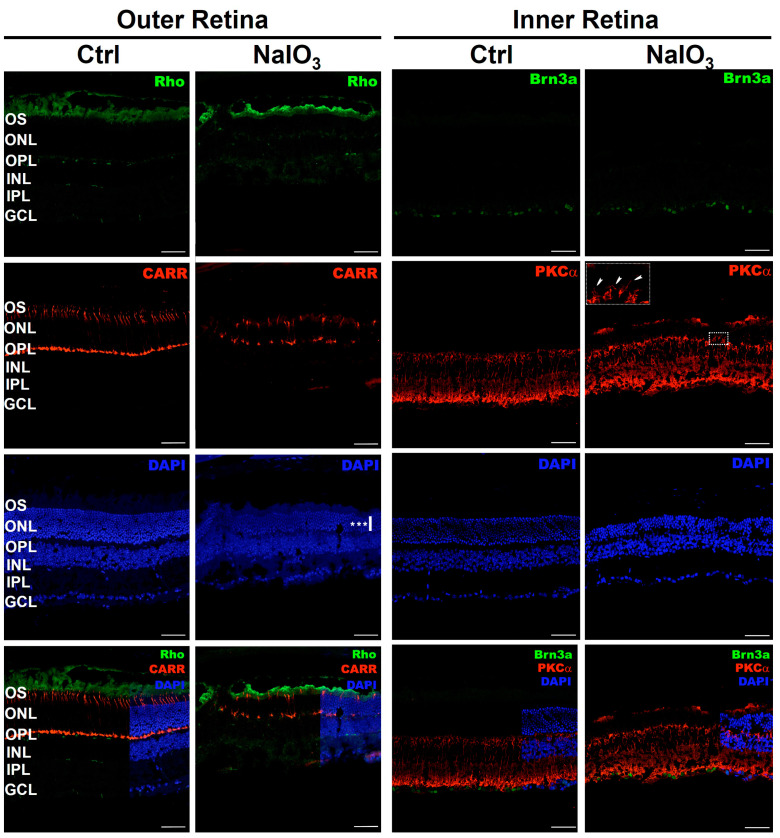
Structural study of the retina after NaIO_3_ injection. Left columns: Labeling of Outer Retinal cells. Images of retinal sections show the labeling of Rhodopsin (Rho, Green) (opsin located in the outer segments of rods) and Cone Arrestin (CARR, Red) (cone-specific marker). Right columns: Labeling of Inner Retinal cells. Images of retinal sections show the labeling of Protein Kinase C alpha (PKCα, Red) (structural marker of bipolar cells), Brn3a (Green) (protein localized in retinal ganglion cells). The intercalating agent DAPI (Blue) was used as a nuclear marker. The insert image of the PKCα labeling of the NaIO_3_ injected animal corresponds to an amplification of the dashed rectangle, showing the dendritic projections from the bipolar cells that extend toward the ONL (arrowheads). The white and vertical bar on DAPI image show a decrease on the ONL. OS, outer segments; ONL, outer nuclear layer; OPL, outer plexiform layer; INL, inner nuclear layer; IPL, inner plexiform layer; GCL, ganglion cell layer. The scale bar represents 75 µm in all micrographs. A significant decrease of the ONL thickness was observed (***, *n* = 3, *p* < 0.001).

At 57 days after NaIO_3_ administration, severe structural damage was observed in the outermost layers of the retina (see Figure 7). Almost complete degeneration of the outer segments of the rods (labeled with rhodopsin: Rho) was observed. Similarly, the cones showed significant retraction in their structure (labeled with cone arrestin: CARR). Likewise, DAPI labeling of nuclei showed a slight decrease in the number of photoreceptor nuclei. These data demonstrate that the Outer Nuclear Layer (ONL) was severely damaged by the systemic administration of NaIO_3_.

Labeling of bipolar cells (using antibodies to PKCα) and ganglion cells (using antibodies to Brn3a) showed no apparent structural damage in the inner retinal layers. However, dendritic projections from the bipolar cells were observed to extend beyond the Outer Plexiform Layer (OPL), perhaps in search of maintaining synapses with the collapsing photoreceptors (see insert in Figure 7).

Measurements of the retinal layers (Table 2) indicated a marked decrease in the thickness of the ONL compared to the control group (*n* = 3, *p* < 0.001), while the INL was not affected (*n* = 3, *p* < 0.224). Differences were also observed in the number of nuclei per row in the ONL (*n* = 3, *p* < 0.001), while there was no variation in number of nuclei per row of the INL (*n* = 3, *p* < 0.224).

**Figure 8:**
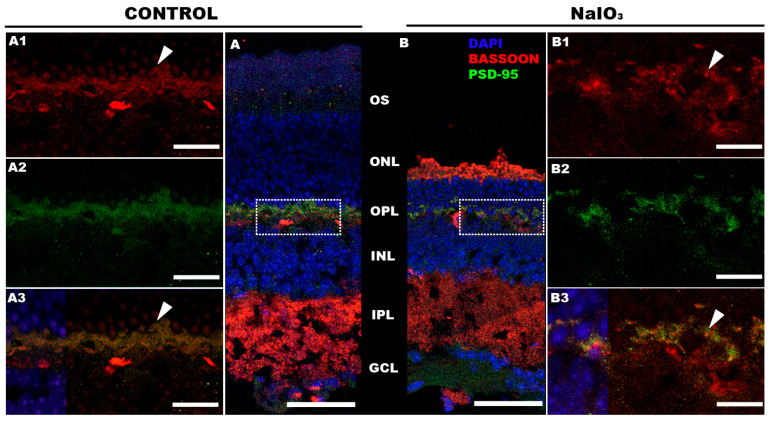
Synapses between photoreceptor and second-order neurons were maintained after NaIO_3_ injection. Bassoon (red, protein located in the rod’s presynaptic terminal) and PSD-95 (green, protein located in the postsynaptic zone) labeling is shown. Immunohistochemical retinal labeling of an animal of the control group (**A**) and NaIO_3_-injected group (**B**). (**A1**,**B1**) Bassoon labeling. (**A2**,**B2**) PSD-95 labeling. (**A3**,**B3**) Merge. The arrowheads show an example of the Basson surrounded by PSD-95 proteins in both groups. The intercalating agent DAPI (Blue) was used as a nuclear marker. The scale bars represent: (**A**,**B**) 50 µm; (**A1**–**B3**) 10 µm.

A study of the synaptic processes between photoreceptors and postsynaptic cells was also carried out (see Figure 8). Apparently, no significant alterations of presynaptic (Bassoon) or postsynaptic (PSD-95) proteins were observed after NaIO_3_ administration.

## 4. Discussion

Sodium iodate has previously been used to induce oxidative stress damage in the RPE, simulating the effects of AMD [12]. Thus, the injury first causes RPE cell death, followed by progressive photoreceptor death [16,17,27,41]. Based on previous studies [17,42,43,44,45], this study characterized the degree of visual impairment in mice in which retinal damage was induced by intraperitoneal administration of a single dose of 65 mg/kg sodium iodate. To prevent other photosensitive retinal cells (intrinsically photosensitive Retinal Ganglion Cells, iPRGCs) from generating responses to light stimulation, animals with a mutation in the melanopsin gene (*Opn4^−/−^*) were used, disabling the photosensitivity of iPRGCs [32]. The animals were subjected to behavioral and non-invasive tests to study their responses to light stimuli.

The light/dark transition test explores mice’s aversion to light. In our experiments, the transition test addressed the difficulty for the animal injected with sodium iodate to detect the illuminated space. At 14 days after NaIO_3_ injection, the time spent by the animals in the dark box was significantly less than the control group. At 28 days after injection, animals spent similar times in the illuminated and dark boxes. These results are similar to those shown by other authors [46], although they opted for intravenous rather than intraperitoneal administration of sodium iodate [17]. 

The impairment of visual acuity after sodium iodate injection was one of the first signs to manifest itself, as 3 days after injection, mice suffered a marked loss, and at 14 days, they were unable to distinguish different visual stimuli, as shown by the optomotor test. These results agree with those reported in the previously published work [17,46,47,48], where the various groups suffered a rapid loss of visual acuity after the first week. However, it should be noted that these groups showed slight improvements in visual acuity after a period. In our study, no such improvement was observed.

Analysis of non-imaging visual pathways, such as the pupillary reflex, showed that they were severely affected 3 days after injection. This reflex starts with the involvement of photoreceptors and melanopsin ganglion cells [49]. Since the mice used in this model had a mutation in the melanopsin gene (*Opn4^−/−^*) that canceled its photosensitive function, the responses obtained in this reflex after the injection of sodium iodate corresponded exclusively to the photoreceptors, and their decrease was a faithful reflection of the lesion they suffered [40]. The novelty of these experiments is that their results reinforce the correlation with pupillary reflex dysfunction observed in patients suffering from various retinal disorders [50,51,52,53]. However, further research would be required to discern the origin of this dysfunction.

In synchrony with the analysis of the optomotor test and the pupillary reflex, electroretinography showed a great affectation of the pure scotopic responses of the ERG in the early stages (day 3); at 7 days, a greater decrease in amplitude was observed, being very difficult to register after 14 days. Of particular interest is the fact that cone degeneration was slower than rod degeneration, which was noticeable because the loss of photopic b-wave functionality occurred in a more graded manner.

Some studies have shown that after initial functional retinal damage with sodium iodate, there is a very pronounced recovery of a- and b-waves within 24 h of administration [54,55]. However, 3 days after injection, a pronounced decrease in a- and b-waves was observed under both scotopic and photopic conditions [12,17,22]. Subsequently, after 7 days, the decrease in the amplitude of scotopic and photopic a- and b-waves was much greater, consistent with previous studies [17,22,44,56]. Similar results have also been observed in rats [42,57]. It is therefore clear that there is extensive functional damage to the photoreceptors, which prevents phototransduction and transmission of the visual stimulus to the inner retina. In addition, it was observed that the cones are more resistant to indirect damage caused by the loss of RPE cells than the rods, degenerating more slowly.

The study of the impact of sodium iodate administration on the function of the visual cortex showed a significant decrease in the responses recorded by the VEP compared to the control group, which showed robust responses very similar to those of other studies [58,59]. Of particular interest is the fact that this reduction is not proportional to that observed in the retina for that analysis time. Thus, despite the complete disappearance of the ERG waves, it was possible to record the characteristic VEP components, albeit with a lower amplitude, as was also reflected in other studies performed in rats [60]. This phenomenon may be caused by amplification mechanisms in the visual cortex. Thus, the photosensitive cells remaining after neurodegeneration transmit the information to the visual cortex, where it is amplified [61,62,63]. These data suggest that the observed abnormalities in the response of the visual cortex are closely associated with NaIO_3_-induced retinal alterations, since no damage to other structures of the visual pathway (lateral geniculate nucleus or visual cortex) has been described [59].

Sodium iodate is known to cause necrosis in RPE cells, which, in turn, triggers a series of cell death mechanisms in photoreceptors [64,65]. A structural study of the retina showed that the lesion caused a strong disorganization of the Outer Nuclear layer (ONL), resulting in invaginations formed by rows of nuclei [66,67,68]. These results were corroborated by Optical Coherence Tomography (OCT) [20]. The lesions in the ONL layer showed a maximum peak of appearance at 7 and 14 days [14,20,69]. In addition, a clear decrease in the thickness of the outer retina was observed after administration of the compound [17,22,26,68]. Whereas the retinal structural damage induced by sodium iodate has been well characterized up to 28 days [17], there is little information on the morphological state of the retina at later stages of neurodegeneration (57 days). At this post-administration time, a strong involvement of cones and rod outer segments was found. In general, a disorganization of the ONL and a shortening of the cones could be observed, although without complete degeneration. These results are like those observed in previously published work [17]. Regarding the inner retina, no clear cell damage was observed that could compromise its function. However, from day 14 after sodium iodate administration, dendritic processes from the bipolar cells toward the outer retina were detected. It could be assumed that the loss of synaptic contact caused by photoreceptor death induces this growth in the dendrites of the bipolar cells with the intention of reconnecting with the synaptic terminal of the cones and rods. Paradoxically, it has been reported that after injury, many of the bipolar cells undergo atrophy of their dendritic processes [28]. In any case, further studies would be necessary to clarify the alterations in the dendrites of bipolar cells. Interestingly, the number of remanent nuclei in the ONL does not seem to agree with the results obtained by the ERG. Therefore, it was decided to study the synaptic processes between photoreceptors and postsynaptic cells. Apparently, the labeling did not show severe synapse damage at the OPL. This could indicate that the loss of retinal functionality is specifically directed to the degeneration of photoreceptor outer segments, following the death of the pigment epithelium by NaIO_3_ [15,17].

Our work demonstrates that, because of the death of the pigment epithelium cells and subsequently of the photoreceptors, the process of phototransduction is greatly impeded, and visual information is prevented from being transmitted from the retina to the higher centers of the CNS.

Any experimentation involving this model must consider several features that may limit its use. Among them are the vascular alterations that the retina may undergo, especially in the choriocapillaris layer [56,70,71,72], which are generated by the accumulation of degenerated cellular debris. In addition, destruction of the RPE disrupts the blood–brain barrier. Also, the systemic administration of this compound can cause damage to other organs or tissues of the animal, being lethal at a dose of 100 mg/kg [17]. Finally, it should be noted that, as in other neurodegenerative diseases, in early stages of the process, the variability in loss is more heterogeneous. However, in advanced stages (28 days), the decrease in the amplitude of ERG signals is practically complete. It should also be noted that higher doses reduce the degeneration time and potentially cause other systemic conditions, such as liver or renal failure [24,48].

The applicability of similar models has been successfully demonstrated in certain regeneration experiments, such as the implantation of photoreceptors and RPE cells [46] and human mesenchymal stem cells [44]. The nature of the degeneration induced by sodium iodate makes it a good tool for the study of pathologies such as AMD [8,11], as well as for the study of the mechanisms of cell death triggered by oxidative stress [73] and the evaluation of treatments against degeneration and cell death in the retina due to oxidative stress [74,75,76]. Other studies have shown that the activation of genes related to cellular metabolism produced a beneficial effect in models of geographic atrophy induced by NaIO_3_ administration, promoting photoreceptor survival [77].

One of the most relevant points of this animal model is the application of an oxidative stress agent on a mouse without ipRGC-mediated photosensitivity. In this way, any bias produced by these cells was avoided [3,32,49].

## 5. Conclusions

The visual functions of the animal model of retinal neurodegeneration induced by the administration of sodium iodate (NaIO_3_) combined with a knockout mutation of the *Opn4* gene (*Opn4^−/−^*) have been extensively characterized. NaIO_3_ administration caused severe structural and functional damage to the retina of these animals, which lost virtually all visual functions despite maintaining a small amount of electrical activity in the primary visual cortex. The model presented in this study can be used in the future as a powerful tool for the study of new therapies or pharmacological approaches for the prevention or treatment of retinal pathologies mediated by oxidative stress.

## Figures and Tables

**Table 1:** (**A**) Primary and (**B**) secondary antibodies used in the labeling of retinal cells.

**(A) Primary Antibodies Table**						
**Primary Antibodies**	**Type**	**Host**	**Concentration**	**Provider**	**Labeling**	**RRID Number**
Anti-Cone Arrestin	Polyclonal	Rabbit	1:10,000	Merk-Millipore	Cones	AB_1163387
Anti Rhodopsin	Monoclonal	Mouse	1:200	Merk-Millipore	Rod Outer Segments	AB_2178961
Anti-PKCα	Polyclonal	Rabbit	1:1000	SIGMA	Bipolar Cells	AB_477345
Anti-Brn3	Polyclonal	Goat	1:200	Quimigen (Santa Cruz)	Retinal Ganglion Cells	AB_2167511
Anti-Bassoon	Monoclonal	Mouse	1:400	BIONOVA	Photoreceptors Presynaptic Zone	AB_2313990
Anti-PSD-95	Polyclonal	Rabbit	1:200	Abcam	PSD-95	AB_444362
**(B) Secondary Antibodies Table**						
**Secondary Antibodies**	**Fluorochrome**	**Host**	**Concentration**	**Provider**	**Color**	**RRID Number**
Goat Anti-IgG	Cy^TM^2	Donkey	1:200	VITRO (Jackson)	Green	AB_2307341
Mouse Anti-IgG	Cy^TM^2	Chicken	1:700	VITRO (Jackson)	Green	AB_2535786
Rabbit Anti-IgG	Cy^TM^3	Donkey	1:200	VITRO (Jackson)	Red	AB_2307443

**Table 2:** Morphometric characteristics of the retina from the control and NaIO_3_-injected animals. The ONL underwent a decrease in thickness in the experimental group (*n* = 6) due to damage to the photoreceptors induced by NaIO_3_ administration. Significance was represents as: ns, not statistically significant; **, *p* < 0.01; ***, *p* < 0.001.

	Nucleus		Retinal Thickness			Thickness Ratio	
	ONL	INL	Total Thickness	ONL Thickness	INL Thickness	ONL/Total	INL/Total
Control (*n* = 3)	10.00 (0.62)	5.53 (0.30)	170.42 (13.10)	55.17 (5.02)	41.88 (5.74)	0.324 (0.018)	0.245 (0.019)
NaIO_3_ (*n* = 6)	4.67 (1.20)	5.22 (0.38)	136.77 (3.39)	33.20 (4.93)	37.18 (1.23)	0.243 (0.042)	0.272 (0.002)
*p*-value	<0.001 (***)	0.224 (ns)	0.005 (**)	<0.001 (***)	0.224 (ns)	0.008 (**)	0.066 (ns)

## Data Availability

The data are contained within the article.
